# Effects of GH L127V and TG5 C422T polymorphisms on the hormonal profile, slaughter traits, and meat quality of Hereford bulls

**DOI:** 10.14202/vetworld.2024.1920-1927

**Published:** 2024-08-27

**Authors:** K. M. Dzhulamanov, N. P. Gerasimov

**Affiliations:** Federal Research Center of Biological Systems and Agrotechnologies RAS, Orenburg, Russia

**Keywords:** amino acid, carcass traits, fatty acid, growth hormone gene, Hereford breed, hormones, polymorphism, thyroglobulin gene

## Abstract

**Background and Aim:**

The creation of objective methods for the evaluation and improvement of quantitative and qualitative indicators of meat productivity in farm animals should be based on a comprehensive analysis of the genetic, physiological, and biochemical parameters of the animal. This study aimed to investigate the effects of growth hormone (GH) and thyroglobulin (TG5) gene polymorphisms on the hormonal status, slaughter traits, and chemical, amino acid, and fatty acid composition of meat in Hereford bulls.

**Materials and Methods:**

Hereford bulls (n = 9) were reared under the same feeding and housing conditions until the age of 21 months, after which they were slaughtered. Polymerase chain reaction-restriction fragment length polymorphism was performed for genotyping GH L127V and TG5 C422T polymorphisms. The experimental animals were evaluated to determine slaughter traits (including pre-slaughter weight, carcass, and internal fat weight and yield), chemical, fatty acid, and amino acid composition of ground beef, and hormonal status using serum concentrations of GH, triiodothyronine, and thyroxine.

**Results:**

Animals with the valine homozygous (VV) genotype of GH had the maximum serum GH level of 9.33 mIU/mL (p = 0.10) higher than leucine homozygous (LL) genotype carriers. Individuals with the LL genotype outperformed V-allele carriers in serum thyroxine (T4) concentration by 21.3–30.5 nmol/L (16.15%–24.86%; p < 0.01–0.05). Genetic differentiation induced by TG5 C422T polymorphism was determined to a lesser extent by the hormonal status of the Hereford animals. The V-allele was associated with increased carcass weight, with VV homozygotes significantly outperforming LL individuals by 45.0 kg (13.61%; p < 0.05). The T allele at the TG5 gene polymorphism was associated with more intense lipogenesis and less protein synthesis in muscle tissue and these effects were enhanced in the homozygous state. Young animals with the TT variant of the TG5 gene exhibited a significantly superior polyunsaturated fatty acid/saturated fatty acid ratio of 0.012 units (p < 0.01). Carriers of the LL genotype were characterized by minimum amino acid content in muscle tissue. Heterozygous bulls exceeded LL homozygotes in the sum of essential amino acids by 3.09% (p = 0.10) and non-essential amino acids by 1.9% (p < 0.05).

**Conclusion:**

The development of breeding programs for the Hereford breed should be carried out considering genetic features that determine the formation of economic traits in animals. Analysis of polymorphisms in the TG5 gene is a promising method for the early diagnosis of the fatty acid composition of beef. Identification of polymorphisms in the GH gene allows the prediction of higher productivity potential and amino acid composition of meat. The different effects of the GH and TG5 genes on the development of various economic traits allowed us to determine further vectors for scientific research on their complex associations in Hereford cattle, which will be useful for planning effective breeding schemes.

## Introduction

Among the priority tasks of agricultural development, improving the domestic gene pool of beef cattle breeds is crucial for ensuring a country’s food security and implementing an import substitution program [1]. The creation of highly efficient specialized beef cattle breeding should be focused on intensifying the selection of carriers of desirable genotypes associated with adaptive and productive traits, on this basis creating reference populations and their expansion to whole regions using modern biotechnological methods of reproduction [2]. In this context, studying the genomes of beef cattle based on highly polymorphic genetic markers of economically valuable traits is of great national and state importance. The improvement of breeding resources is aimed at the formation of such populations that, under specific natural and economic conditions, exhibit maximum productivity with the effective use of available resources. Selection and breeding are focused on maintaining and improving the inbreed structure through the intensive use of outstanding animals in the reproductive system. To accelerate selection in beef cattle breeding, it is necessary to complete herds with high-quality animals that exceed the genetic potential of the previous generation. In this case, it is economically feasible to evaluate and select promising young animals at the early stages of development, which implies the widespread implementation of predicting the breeding value of animals based on the identification of functional polymorphisms in genes associated with the formation of meat productivity and beef quality [3, 4]. The annual selection effect of targeting “desirable” genotypes increases by 15%–30% compared with classical selection techniques [5]. Genotyping, a method of comprehensive study of individual genotypes, is included in the selection system of beef cattle breeding [6]. It is currently used to confirm the authenticity of the origin of breeding animals, to identify carriers of hereditary diseases, and to perform marker-assisted selection for genes associated with economically useful traits in beef cattle. Growth hormone (GH) gene polymorphisms are associated with growth and development variability and quantitative and qualitative measures of meat productivity. Nucleotide substitutions in the thyroglobulin (TG5) gene sequence are associated with bovine lipid metabolism traits [7].

The creation of objective methods for evaluating and improving quantitative and qualitative indicators of meat productivity in farm animals should be based on a comprehensive analysis of the physiological and biochemical parameters of the organism [8]. The significant diversity of food products on the market, together with increased consumer attention to the organoleptic properties of products, stimulates the selection of livestock, taking into account the biological and nutritional value of meat [9]. The possibilities for the lifetime determination of meat quality are very limited, but they are highly demanded in livestock breeding. An important resource in this regard is the study of the genetic aspects of morphogenesis of individual tissues, which are closely related to the biochemical composition of the body and, therefore, to the formation of economically useful traits in beef cattle [10]. Increasing the efficiency of beef cattle breeding and intensive use of animals brings to the fore the requirements for resistance and adaptation of the organism; the formation of which depends on the hormonal status, including the influence of thyroid and GHs on metabolism and the functioning of organs and tissues, which ultimately affect the level of meat productivity [11]. Successful development of methods to improve and predict meat productivity requires not so much monitoring of specific biochemical parameters, but rather comprehensive control that combines key metabolic indicators that are physiologically and functionally closely related to each other and represented by a single metabolic system [12]. The implementation of complex tests and the creation of biochemical and genetic markers for use in breeding work in animal husbandry will increase the accuracy of the assessment of productive potential and the lifetime prediction of meat quality [13].

The aim of this study was to investigate the effects of GH and TG5 gene polymorphisms on the hormonal status, slaughter traits, and chemical, amino acid, and fatty acid composition of meat in Hereford bulls.

## Materials and Methods

### Ethical approval

Maintenance of animals and experimental studies were carried out in accordance with the instructions and recommendations of normative acts: Model Law of the Interparliamentary Assembly of the Member States of the Commonwealth of Independent States “On the Treatment of Animals,” Art. 20 (Resolution of the MA of the CIS Member States No. 29-17 dated 31.10.2007), Guidelines for work with laboratory animals (http://fncbst.ru/?page_id=3553). Extracts from the minutes of the meeting of the Commission on the control of the maintenance and use of laboratory animals (Ethics Committee) No. 7 dated January 17, 2023, and received on March 20, 2024. The study was conducted to minimize animal suffering and reduce the number of subjects. The animals were slaughtered according to the method specified in State Standard R 34120-2017 [14].

### Study period and location

The study was conducted from May 2022 to February 2024. The animals were reared at the test station of the “Agrofirma Kalininskaya” LTD (Chelyabinsk Region, Russia). The laboratory tests were conducted at the collaborative center of the Federal Research Center for Biological Systems and Agrotechnologies RAS.

### Animals and sampling

The animals of the study were bulls (n = 9) of the Hereford breed. Bulls of different genotypes were reared under the same feeding and housing conditions until the age of 21 months, after which they were slaughtered. An average of 400 g of ground beef was taken from the left half of each carcass. From the same carcass before deboning, a sample (200 g) of the *longissimus dorsi* muscle at the level of the 9^th^_–_11^th^ ribs was taken by transverse muscle cutting.

Blood (approx. 5 mL) was collected before slaughter in vacuum tubes containing a coagulation activator (SiO_2_). The biomaterials were analyzed using an automated biochemical analyzer (DIRUI CS-T420 (Dirui Industrial Co., China) and an automated microplate analyzer (Infinite F200 PRO (Tecan Austria GmbH, Austria).

Dry matter was determined by drying the samples in a desiccator at 100°C. Organic matter was determined by ashing the dried samples at 550°C [15]. Capillary electrophoresis was performed using a Kapel-105M analyzer (Russia) to study the amino acid composition of beef proteins. The fatty acid composition of meat lipids was determined by gas-liquid chromatography on a chromatograph “Crystal-2000 M” (Russia).

### Genotyping

Blood was collected from the jugular vein of experimental bulls after weaning (8 months of age) after weaning to genotype by polymorphisms of the GH L127V and TG5 C422T genes. DNA extraction was performed with “DIAtom™ DNA Prep” reagents (IsoGeneLab, Russia). Polymerase chain reaction (PCR)-restriction fragment length polymorphism was performed using “GenePakPCRCore” kits (IsoGeneLab) on a programable thermocycler “Tertsik” (DNA-Technology, Russia). Primers (Litech, Russia) were used to amplify the sites. The PCR conditions are described in Table-1.

**Table-1:** Primer sequence, PCR conditions, and restriction enzymes used for genotyping.

SNP	Primer	PCR conditions	Restriction enzymes
GH C2141G	F: 5’- GCTGCTCCTGAGCCTTCG-3’ R: 5’- GCGGCGGCACTTCATGACCCT-3’	Initial heating at 95°°C for 5 min; 35 cycles: denaturation at 94°°C for 45 s; annealing at 65°°C for 45 s; synthesis at 72°°C for 45 s; primer extension at 72°°C for 7 min	*AluI*
TG5 C422T	F: 5’- GGGGATGACTACGAGTATGACTG-3’ R: 5’- GTGAAAATCTTCTGGAGGCTGTA-3’	Initial heating at 94°°C for 4 min; 35 cycles: denaturation at 94°°C for 60 s; annealing at 62°°C for 60 s; synthesis at 72°°C for 60 s; primer extension at 72°°C for 4 min	*BstX2I*

SNP=Single nucleotide polymorphism, PCR=Polymerase chain reaction, GH=Growth hormone, TG5=Thyroglobulin

**Figure-1:**
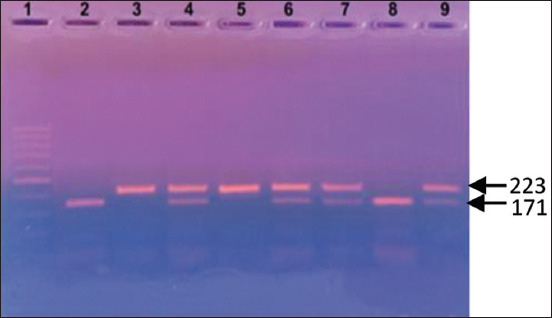
Electrophoretic polymerase chain reaction product visualization of the growth hormone gene in agarose gel. Lane 1: marker with 50-bp DNA ladder (IsoGeneLab, Russia); lane 2, 8: Number of samples with LL genotype (171, 52 bp); lane 4, 6, 7, 9: Number of samples with LV genotype (223, 171, 52 bp); lane 3, 5: number of samples with VV genotype (223 bp).

**Figure-2:**
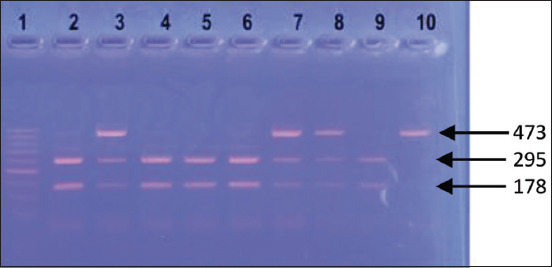
Electrophoretic polymerase chain reaction product visualization of the TG5 gene in agarose gel. Lane 1: Marker with 50-bp DNA ladder (IsoGeneLab, Russia); lane 2, 4, 5, 6, 9: Number of samples with CC genotype (295, 178, 75 bp); lane 3, 7, 8: Number of samples with CT genotype (473, 295, 178, 75 bp); lane 10: Number of samples with TT genotype (473, 75 bp).

The resulting products were separated by horizontal electrophoresis in a 1× Tris-borate buffer at 80 V in 2.5% agarose gel for 30 min and stained with ethidium bromide. The gel was visualized using a “UVT-1” transilluminator and photographed using the “VITran v.1.0” (“Biokom”, Russia). Fragment length was determined using the “GenePakR DNA Ladder M 50” (IsoGeneLab) molecular weight size marker (Figures-1 and 2).

### Statistical analysis

The effects of genotype on the traits studied were analyzed using the least-squares method and the general linear model procedure in Statistica 10.0 software (“Stat Soft Inc.,” USA).

Model used:

Y_ij_=μ+A_i_+e_ij_,

where, Y_ij_ – represents the studied traits, μ – is the overall mean, A_i_ is the fixed effect of the GH and TG5 genotypes (1, 2, 3), and e_ij_ is random error.

The significance of intergenotype differences was assessed using the posteriori Fisher’s criterion (F-test). p ≤ 0.05 was considered statistically significant.

## Results

Hereford bulls were genotyped for GH and TG5 after weaning from cows at 8 months of age. Among the bulls of the herd (n = 50), the GH L127V polymorphism was represented by two alleles, L and V, with frequencies of 0.66 and 0.34, respectively, forming the three genotypes LL, LV, and VV, with proportions of carriers of 46, 40, and 14%, respectively. A similar frequency of alternative alleles was found for the TG5 C422T polymorphism, which was 0.65 for the C allele and 0.35 for the T allele. The number of carriers of the CC genotype was 38%, CT - 54%, and TT - 8%.

The concentration of endogenous GH varied widely in Hereford bulls with different genotypes of GH L127V polymorphism, ranging from 7.91 to 17.24 mIU/mL (Table-1). Animals with the LL genotype had minimal serum GH levels, which were 7.05 mIU/mL (p = 0.20) lower than those of heterozygous bulls and 9.33 mIU/mL (p = 0.10) lower than those of VV genotype carriers. The dynamics of thyroid hormones were also determined by genetic differences in young animals with respect to GH expression. Thus, heterozygous bulls were characterized by elevated triiodothyronine (T3) levels of 0.14–0.21 nmol/L (6.03%–9.33%; p > 0.05) compared with their homozygous counterparts. In turn, individuals with the LL genotype outperformed V-allele carriers in serum thyroxine (T4) concentration by 21.3–30.5 nmol/L (16.15%–24.86%; p < 0.01–0.05).

Genetic differentiation of Hereford cattle by TG5 C422T polymorphism determined to a lesser extent the hormonal status of the animals. CC-genotype bulls outperformed their counterparts in endogenous GH concentration by 1.02–3.80 mIU/mL (7.31%–33.99%; p > 0.05) but were inferior to T-allele carriers in triiodothyronine content by 0.12–0.22 nmol/L (5.11%–8.98%; p > 0.05) and thyroxine by 9.7–18.7 nmol/L (7.12%–12.88%; p > 0.05). Thus, despite the genetic peculiarities of young animals, no significant deviations were observed in the synthesis of the products of the genes studied.

Carriers of the V-allele of the GH gene showed a high intensity of weight growth during the fattening period, which was expressed in superiority in pre-slaughter weight of 43.0–63.0 kg (7.60%–11.13%; p > 0.05) compared with bulls with the LL-genotype (Table-2). The V-allele was also associated with increased carcass weight in Hereford cattle, with VV homozygotes significantly outperforming LL individuals by 45.0 kg (13.61%; p < 0.05). The same rank of genotype distribution for GH L127V polymorphism was observed for carcass yield. A more intense process of internal fat accumulation was observed in heterozygous bulls, which outperformed their homozygous counterparts in terms of weight by 0.7–1.2 kg (4.40%–7.79%; p > 0.05) and fat yield (0.2% (p > 0.05).

**Table-2:** Effect of GH and TG5 gene polymorphisms on endogenous hormone dynamics in the blood plasma of Hereford bulls (M ± SE).

Hormone	The GH genotype			The TG5 genotype		
	
	LL	LV	VV	CC	CT	TT
Growth hormone (mIU/mL)	7.91 ± 2.23	14.96 ± 4.45	17.24 ± 3.27	14.98 ± 4.86	11.18 ± 3.51	13.96 ± 4.39
Triiodothyronine, nmol/L	2.32 ± 0.03	2.46 ± 0.13	2.25 ± 0.07	2.23 ± 0.05	2.45 ± 0.13	2.35 ± 0.01
Thyroxine, nmol/L	153.2 ± 5.79^ab^	131.9 ± 4.51^a^	122.7 ± 6.24^b^	126.5 ± 6.10	136.2 ± 14.35	145.2 ± 2.51

a Values in a row with the same indexes differ with significance p < 0.05,

b p < 0.01. GH=growth hormone gene, TG5=Thyroglobulin, SE=Standard Error

TG5 C422T polymorphism had no significant effect on the variability of slaughter traits in Hereford bulls. Carriers of the CC genotype had the highest values for all slaughter traits, whereas heterozygous animals had the lowest.

Despite insignificant differences in the chemical composition of meat due to polymorphisms of GH and TG5, it is necessary to note the existing trends in the accumulation of nutrients in the carcasses of bulls of different genotypes (Table-3). The LL genotype was associated with increased fat accumulation by 1.11%–1.42% and minimal protein synthesis by 0.82%–1.25% compared to V allele carriers. On the other hand, heterozygous bulls were characterized by high protein and low-fat content.

**Table-3:** Effects of GH and TG5 gene polymorphisms on the slaughter performance dynamics of Hereford bulls (M ± SE).

Indicator	The GH genotype			The TG5 genotype		
	
	LL	LV	VV	CC	CT	TT
Pre-slaughter weight (kg)	566.0 ± 24.03	609.0 ± 23.46	629.0 ± 5.51	607.7 ± 22.51	597.3 ± 26.77	599.0 ± 30.66
Carcass weight (kg)	330.7 ± 13.72^a^	363.0 ± 16.09	375.7 ± 6.36^a^	364.7 ± 16.70	350.3 ± 17.75	354.3 ± 19.43
Carcass yield, %	58.4 ± 0.34	59.6 ± 0.36	59.7 ± 0.80	60.0 ± 0.55	58.6 ± 0.61	59.1 ± 0.50
Internal fat mass (kg)	15.4 ± 0.49	16.6 ± 0.91	15.9 ± 1.08	16.7 ± 0.78	15.2 ± 0.57	16.0 ± 1.04
Internal fat yield, %	2.73 ± 0.15	2.73 ± 0.17	2.53 ± 0.19	2.76 ± 0.19	2.56 ± 0.22	2.66 ± 0.09

a Values in a row with the same indexes differ with significance p < 0.05. GH=Growth hormone gene, TG5=Thyroglobulin, SE=Standard error

T allele at the TG5 gene polymorphism was associated with more intense lipogenesis and less protein synthesis in the muscle tissue of Hereford bulls, and these effects were enhanced in the homozygous state. Thus, the difference in meat fat content reached 1.38% and protein 0.68% between two alternative homozygous genotypes. Intermediate nutrient accumulation in carcass meat was observed in heterozygous animals.

The genetic characteristics of young animals with GH gene polymorphisms had no significant effect on the fatty acid composition of beef (Table-4). A trend toward higher polyunsaturated fatty acid (PUFA) content [0.44% (p = 0.055)] and lower saturated fatty acid content [0.46% (p > 0.05)] was observed in carriers of the LL genotype compared with the alternative homozygous variant of the gene. This result was reflected in a higher PUFA/saturated fatty acid (SFA) ratio of 0.010 units (p = 0.06). The PUFA advantage in steers with the LL variant of the GH gene was due to increased synthesis of linoleic acid by 0.20%–0.30% (p = 0.10) and linolenic acid by 0.06%–0.13% (p > 0.05) compared with their counterparts.

**Table-4:** Effects of GH and TG5 gene polymorphisms on the chemical composition of meat in Hereford bulls (M ± SE), %

Indicator	The GH genotype			The TG5 genotype		
	
	LL	LV	VV	CC	CT	TT
Moisture	69.94 ± 1.11	70.10 ± 1.81	70.22 ± 1.15	70.62 ± 1.31	69.94 ± 1.10	69.70 ± 1.65
Fat	11.06 ± 1.74	9.64 ± 1.56	9.95 ± 1.04	9.44 ± 1.24	10.38 ± 1.20	10.82 ± 1.91
Protein	18.11 ± 0.79	19.36 ± 0.27	18.93 ± 0.14	19.03 ± 0.11	19.02 ± 0.45	18.35 ± 0.87
Ash	0.89 ± 0.02	0.90 ± 0.02	0.90 ± 0.01	0.91 ± 0.01	0.89 ± 0.01	0.89 ± 0.02

GH=growth hormone gene, TG5=Thyroglobulin, SE=Standard error

Variability in the fatty acid composition of Hereford bull meat was determined by polymorphisms of the TG5 gene. The T allele is associated with an increased and decreased content of unsaturated FAs in the lipid structure of muscle tissue. Moreover, when the allele is homozygous, these effects increase. Thus, CC genotype carriers had 0.46%–1.53% (p > 0.05) more saturated FAs than their T allele counterparts. The differences between the homozygous genotypes were 1.10% (p > 0.05) for monounsaturated FA and 0.43% (p = 0.06) for polyunsaturated FA. Ranking the genotype distribution on the fatty acid profile of meat provided young animals with the TT variant of the TG5 gene with a significant superiority in the PUFA/SFA ratio of 0.012 units (p < 0.01).

The GH gene polymorphism in Hereford bulls caused a significant difference in the arginine content [0.31% (p < 0.05)] and serine content [0.27% (p < 0.05)] between heterozygous and LL genotypes and in the methionine content [0.29% (p < 0.05)] between homozygous individuals (Table-5). In addition, carriers of the LL variant were characterized by the minimum content of the studied amino acids in muscle tissue. Heterozygous bulls exceeded LL homozygotes in the sum of essential amino acids by 3.09% (p = 0.10) and non-essential amino acids by 1.9% (p < 0.05).

**Table-5:** Effects of GH and TG5 gene polymorphisms on the fatty acid composition of meat in Hereford bulls (M ± SE), %.

Fatty acid	The GH genotype			The TG5 genotype		
	
	LL	LV	VV	CC	CT	TT
C_14:0_	2.67 ± 0.418	3.17 ± 0.273	3.10 ± 0.208	3.20 ± 0.115	2.87 ± 0.441	2.87 ± 0.348
C_16:0_	26.43 ± 0.145	26.97 ± 0.617	26.43 ± 0.296	26.57 ± 0.219	26.80 ± 0.702	26.47 ± 0.120
C_16:1_	3.47 ± 0.145	3.57 ± 0.088	3.63 ± 0.120	3.60 ± 0.115	3.43 ± 0.145	3.63 ± 0.067
C_18:0_	20.07 ± 0.426	19.17 ± 0.689	20.10 ± 0.321	20.27 ± 0.338	19.90 ± 0.608	19.17 ± 0.491
C_18:1_	42.20 ± 0.757	42.30 ± 0.929	42.00 ± 0.404	41.70 ± 0.208	42.03 ± 0.524	42.77 ± 1.004
C_18:2_	3.17 ± 0.120	2.87 ± 0.088	2.97 ± 0.120	2.90 ± 0.153	3.00 ± 0.058	3.10 ± 0.153
C_18:3_	0.53 ± 0.033	0.47 ± 0.033	0.40 ± 0.173	0.43 ± 0.176	0.47 ± 0.033	0.50 ± 0.058
C_20:4_	1.47 ± 0.033	1.50 ± 0.058	1.37 ± 0.120	1.33 ± 0.088	1.50 ± 0.058	1.50 ± 0.058
SFA	49.17 ± 0.524	49.30 ± 1.026	49.63 ± 0.498	50.03 ± 0.186	49.57 ± 0.593	48.50 ± 0.794
MUFA	45.67 ± 0.694	45.87 ± 1.009	45.63 ± 0.393	45.30 ± 0.100	45.47 ± 0.581	46.40 ± 0.985
PUFA	5.17 ± 0.176	4.83 ± 0.067	4.73 ± 0.120	4.67 ± 0.088	4.97 ± 0.067	5.10 ± 0.200
PUFA/SFA	0.105	0.098	0.095	0.093^b^	0.100	0.105^b^

b Values in a row with the same indices differ with significance p < 0.01. SFA=Saturated fatty acid, MUFA=Monounsaturated fatty acid, PUFA=Polyunsaturated fatty acid. GH=Growth hormone gene, TG5=Thyroglobulin, SE=Standard error

There were no significant differences in the amino acid composition of meat among Hereford bulls grouped by TG5 gene polymorphism (Table-6). Young animals with the TT variant were 1.17%–1.28% (p > 0.05) lower in total essential amino acids and 0.53%–0.55% (p > 0.05) lower in non-essential amino acids than heterozygous and homozygous carriers of the C allele.

**Table-6:** Effects of GH and TG5 gene polymorphisms on the amino acid composition of meat in Hereford bulls (M ± SE), %.

Amino acid	The GH genotype			The TG5 genotype		
	
	LL	LV	VV	CC	CT	TT
Arginine	3.34 ± 0.144^a^	3.65 ± 0.052^a^	3.44 ± 0.020	3.47 ± 0.048	3.58 ± 0.082	3.39 ± 0.175
Lysine	4.75 ± 0.354	5.31 ± 0.235	4.96 ± 0.069	5.09 ± 0.059	4.97 ± 0.084	4.96 ± 0.500
Tyrosine	1.80 ± 0.168	2.00 ± 0.075	2.07 ± 0.080	2.02 ± 0.070	2.00 ± 0.084	1.84 ± 0.194
Phenylalanine	2.03 ± 0.164	2.30 ± 0.054	2.30 ± 0.069	2.28 ± 0.072	2.28 ± 0.017	2.08 ± 0.203
Histidine	1.55 ± 0.115	1.75 ± 0.067	1.77 ± 0.039	1.74 ± 0.023	1.73 ± 0.053	1.61 ± 0.159
Leucine+Isoleucine	7.21 ± 0.593	8.02 ± 0.305	7.66 ± 0.212	7.73 ± 0.190	7.75 ± 0.159	7.41 ± 0.748
Methionine	1.33 ± 0.089^a^	1.58 ± 0.049	1.62 ± 0.084^a^	1.53 ± 0.046	1.60 ± 0.118	1.40 ± 0.125
Valine	2.84 ± 0.199	3.21 ± 0.114	3.06 ± 0.079	3.08 ± 0.075	3.07 ± 0.033	2.96 ± 0.286
Proline	2.04 ± 0.310	2.66 ± 0.142	2.50 ± 0.081	2.52 ± 0.068	2.51 ± 0.063	2.17 ± 0.420
Threonine	2.40 ± 0.137	2.70 ± 0.020	2.46 ± 0.084	2.53 ± 0.106	2.57 ± 0.070	2.46 ± 0.176
Serine	2.07 ± 0.097^a^	2.34 ± 0.009^a^	2.16 ± 0.086	2.23 ± 0.100	2.20 ± 0.062	2.14 ± 0.138
Alanine	3.35 ± 0.236	3.77 ± 0.141	3.46 ± 0.046	3.53 ± 0.041	3.55 ± 0.087	3.49 ± 0.339
Glycine	2.83 ± 0.111	3.24 ± 0.179	2.86 ± 0.074	2.93 ± 0.023	2.95 ± 0.156	3.05 ± 0.267
Essential	25.45 ± 1.779	28.54 ± 0.780	22.27 ± 0.350	27.44 ± 0.358	27.55 ± 0.221	26.27 ± 2.370
Non-essential	12.10 ± 0.656^a^	14.00 ± 0.506^a^	13.05 ± 0.145	13.24 ± 0.042	13.22 ± 0.269	12.69 ± 1.200

a Values in a row with the same indexes differ with significance p < 0.05. GH=Growth hormone gene, TG5=Thyroglobulin, SE=Standard error

## Discussion

In our study, we analyzed the effects of the GH and TG5 gene loci on the development of meat productivity and beef quality, as well as hormonal status in Hereford bulls of different genotypes. According to Karisa *et al*. [16], the influence of a quantitative trait locus on the variability of a single breeding trait in beef cattle can be 19.7%. In our experiment, despite noticeable differences in slaughter traits between bulls of different genotypes, nucleotide substitution in the gene had no statistically significant effect on the overall phenotypic variability of traits due to the small number of animals. However, a trend of 47.2% (p = 0.15) for genetic determination of pre-slaughter live weight and 52.5% (p = 0.11) for carcass weight was observed in Herefords. Differentiation of bulls according to genotypes of the GH gene resulted in a significant difference in carcass weight of 13.61% (p < 0.05) between homozygous animals and the formation of an intermediate variant in heterozygous individuals, indicating an additive effect of nucleotide substitution in the gene locus. The effect of GH L127V polymorphism on the variability of slaughter indicators in cattle is noted in the works of many authors [17–20]. However, the association between specific alleles and meat trait development is highly inconsistent. For example, in studies by Lee *et al*. [18] and Plakhtyukova and Selionova [19], carriers of the V allele showed a better ability to weight increase, and more massive carcasses were obtained from them. Sedykh *et al*. [17] and Miroshnikov *et al*. [20] reported the positive effect of the L allele on the expression of slaughter indices in beef cattle. The association between GH L127V polymorphism and carcass traits in beef cattle appears to be breed- and population-specific. Therefore, further research on the relationship between GH expression and slaughter performance is needed to develop breeding programs for beef breeds.

According to Sycheva *et al*. [21], the substitution of the nucleotide sequence C→T in the TG5 gene was not associated with a significant change in carcass performance in Simmental steers. This result is consistent with the slaughter results of Hereford bulls in our studies. Carriers of the CC genotype tended to have an increased carcass yield of 1.4% (p = 0.14) compared with heterozygotes. Ardicli *et al*. [22] observed a significant association between TG5 expression and pre-slaughter and carcass weight formation, with the advantage of the CC genotype relative to the Simmentals of other gene variants. Makaev *et al*. [23] reported that the progeny of Kazakh white-headed sires with the TG5^CC^ genotype outperformed their counterparts in terms of both the absolute weight of adipose tissue and its proportion in the anatomical parts of the carcass. Our studies confirmed the tendency of TG5^CC^ carriers to be superior in the development of these traits in Hereford.

No significant effect on nutrient accumulation in meat was found for polymorphisms of GH and TG5 genes. Differentiation of bulls by GH L127V polymorphism explained 35.87% of the variability in beef protein content and 7.84% of the variability in fat content. The effect of the TG5 C422T gene on the chemical composition of Hereford meat was lower, ranging from 13.40% for protein to 6.94% for fat. However, Dolmatova *et al*. [24] and Sedykh *et al*. [25] found a significant association (p < 0.05) between TG5 gene polymorphism and intramuscular fat content in young Hereford animals. According to their data, carriers of the TT genotype had a better ability to synthesize fat in muscle tissue. In turn, during the fattening of Hereford and Limousin bulls with the LL genotype for the GH gene, increased fat accumulation in the carcasses was observed [17], which was confirmed in our study.

According to Lapshina *et al*. [26], polyunsaturated fatty acid content is negatively correlated with pre-slaughter and carcass weight. In addition, the high intensity of fat accumulation in cattle is associated with changes in the lipid structure of muscle tissue, with increased synthesis of saturated and monounsaturated fatty acids [27]. In our study, carriers of the GH^VV^ genotype with more massive carcasses were characterized by a low PUFA/SFA ratio. Bulls with the TT variant of the TG5 gene combined moderate fat accumulation ability (by weight and internal fat yield) with the maximum PUFA/SFA ratio in meat, which was significantly (p < 0.01) superior to homozygous counterparts by 0.012 units. A significant effect of TG5 gene polymorphism on the variability in serum fatty acid composition was observed in Hereford cattle [28].

## Conclusion

Breeding programs for the Hereford breed should be developed, considering genetic features that determine the formation of economic traits in animals. Analysis of polymorphisms in the TG5 gene is a promising method for the early diagnosis of the fatty acid composition of beef. Identification of polymorphisms in the GH gene allows the prediction of higher productivity potential and amino acid composition of meat. The different effects of the GH and TG5 genes on the development of various economic traits allowed us to determine further vectors for scientific research on their complex associations in Hereford cattle, which will be useful for planning effective breeding schemes.

## Authors’ Contributions

NG and KD: Performed the study and studied scientific literature on the topic. NG: Wrote and edited the article. KD: Designed and conducted the study. Both authors have read, reviewed, and approved the final manuscript.
